# Gene expression signature of Gleason score is associated with prostate cancer outcomes in a radical prostatectomy cohort

**DOI:** 10.18632/oncotarget.17428

**Published:** 2017-04-26

**Authors:** Min A. Jhun, Milan S. Geybels, Jonathan L. Wright, Suzanne Kolb, Craig April, Marina Bibikova, Elaine A. Ostrander, Jian-Bing Fan, Ziding Feng, Janet L. Stanford

**Affiliations:** ^1^ Division of Public Health Sciences, Fred Hutchinson Cancer Research Center, Seattle, WA, USA; ^2^ Department of Epidemiology, GROW School for Oncology and Developmental Biology, Maastricht University, Maastricht, The Netherlands; ^3^ Department of Urology, University of Washington School of Medicine, Seattle, WA, USA; ^4^ Department of Oncology, Illumina, Inc., San Diego, CA, USA; ^5^ Cancer Genetics and Comparative Genomics Branch, National Human Genome Research Institute, NIH, Bethesda, MD, USA; ^6^ Department of Biostatistics, University of Texas MD Anderson Cancer Center, Houston, TX, USA; ^7^ Department of Epidemiology, School of Public Health, University of Washington, Seattle, WA, USA

**Keywords:** gene expression, Gleason score, prostate cancer, recurrence, metastasis

## Abstract

Prostate cancer (PCa) is a leading cause of cancer-related mortality worldwide. Gleason score (GS) is one of the best predictors of PCa aggressiveness, but additional tumor biomarkers may improve its prognostic accuracy. We developed a gene expression signature of GS to enhance the prediction of PCa outcomes. Elastic net was used to construct a gene expression signature by contrasting GS 8-10 vs. ≤6 tumors in The Cancer Genome Atlas (TCGA) dataset. The constructed signature was then evaluated for its ability to predict recurrence and metastatic-lethal (ML) progression in a Fred Hutchinson (FH) patient cohort (N=408; N_Recurrence_=109; N_MLprogression_=27). The expression signature included transcripts representing 49 genes. In the FH cohort, a 25% increase in the signature was associated with a hazard ratio (HR) of 1.51 (P=2.7×10^−5^) for recurrence. The signature's area under the curve (AUC) for predicting recurrence and ML progression was 0.68 and 0.76, respectively. Compared to a model with age at diagnosis, pathological stage and GS, the gene expression signature improved the AUC for recurrence (3%) and ML progression (6%). Higher levels of the signature were associated with increased expression of genes in cell cycle-related pathways and decreased expression of genes in androgen response, estrogen response, oxidative phosphorylation, and apoptosis. This gene expression signature based on GS may improve the prediction of recurrence as well as ML progression in PCa patients after radical prostatectomy.

## INTRODUCTION

Prostate cancer (PCa) is the most common non-cutaneous cancer and a leading cause of cancer-related deaths among men in the U.S. Following primary curative treatment, PCa recurrence rates vary depending on stage, Gleason score (GS), and prostate-specific antigen (PSA) level [1]. Although 20 to 30% of patients with clinically localized disease will relapse within 5 years after initial therapy [2], predicting an individual patient's risk of recurrence or metastatic progression remains challenging.

Several previous studies have constructed prognostic gene expression signatures based on cell cycle proliferation genes [3], prostate tumorigenesis related genes [4], and PCa recurrence [5–7]. In addition, GS has been utilized to construct gene expression signatures [8–10], which have been associated with relapse [8] and prostate cancer mortality [9, 10]. The approach of identifying differentially expressed transcripts in tumors stratified by GS is expected to capture relevant data on tumor aggressiveness potential since GS is one of the best predictors of PCa prognosis [11]. While patients with GS ≤6 tumors typically have a favorable prognosis, patients with GS 8-10 tumors often have a poor prognosis [12]. A substantial proportion of PCa patients, however, have intermediate GS 7 tumors (3+4 or 4+3), and these patients have a more variable prognosis [13]. Better prognostic markers are needed to risk stratify the clinically heterogeneous subset of patients with intermediate GS 7 tumors.

In this study, we developed a GS based gene expression signature by utilizing publically available transcriptome-wide data from The Cancer Genome Atlas (TCGA; N=333) and Elastic net. The constructed signature was then evaluated for its ability to predict recurrence and metastatic-lethal (ML) progression after radical prostatectomy in a PCa patient cohort (N=408, mean follow-up for biochemical recurrence=8 years).

## RESULTS

### Descriptive characteristics

The mean age of the 333 patients in TCGA was 60.6 years (N_missing age_ = 54). The majority of patients was Caucasian (N=270; 81%). The number of patients with tumors classified as GS ≤6, 7 (3+4), 7 (4+3), and 8-10 was 65 (20%), 102 (31%), 78 (23%), and 88 (26%), respectively.

The mean age at diagnosis of the Fred Hutchinson (FH) patients was 58.2 years (Table 1), and most were Caucasian (N=383; 94%). There were 190 (47%), 152 (37%), 34 (8%), and 32 (8%) prostate tumors classified as GS ≤6, 7 (3+4), 7 (4+3), and 8-10, respectively. About 67% (N=273) of patients had local pathological stage while 33% (N=135) had regional pathological stage. Prostate-specific antigen (PSA) levels at diagnosis ranged from 0.8 ng/mL to 66.4 ng/mL with a median of 6.1 ng/mL. Patients in the FH cohort had an average follow-up time for biochemical recurrence of 8.1 years. Patients had recurrence status determined based on follow-up surveys and medical records. During follow-up, 109 recurrence events were identified, including 82 patients with biochemical recurrence and 27 patients who developed metastatic progression or died of their PCa.

**Table 1:** Selected characteristics of prostate cancer patients in the Fred Hutchinson-based radical prostatectomy cohort

*Continuous variables*	N	Mean (SD)
Age at diagnosis (years)	408	58.2 (6.9)
Follow-up time for recurrence (years)	405	8.1 (4.2)
***Categorical variables***	**N**	**Percent**
Race		
African-American	25	6.1%
Caucasian	383	93.9%
Pathological stage^a^		
Local	273	66.9%
Regional	135	33.1%
Gleason score		
≤6	190	46.6%
7(3+4)	152	37.3%
7(4+3)	34	8.3%
8-10	32	7.8%
PSA at diagnosis (ng/mL)		
0-3.9	63	15.4%
4-9.9	231	56.6%
10-19.9	61	15.0%
≥20	27	6.6%
Missing	26	6.4%
Recurrence		
No recurrence	299	73.3%
Biochemical recurrence	82	20.1%
Metastatic-lethal progression	27	6.6%

*PSA*: prostate-specific antigen, SD: standard deviation

^a^ Local stage is pT2, N0/NX, M0. Regional stage is pT3-T4 and/or N1, M0

### Gene expression signature

**Figure 1:**
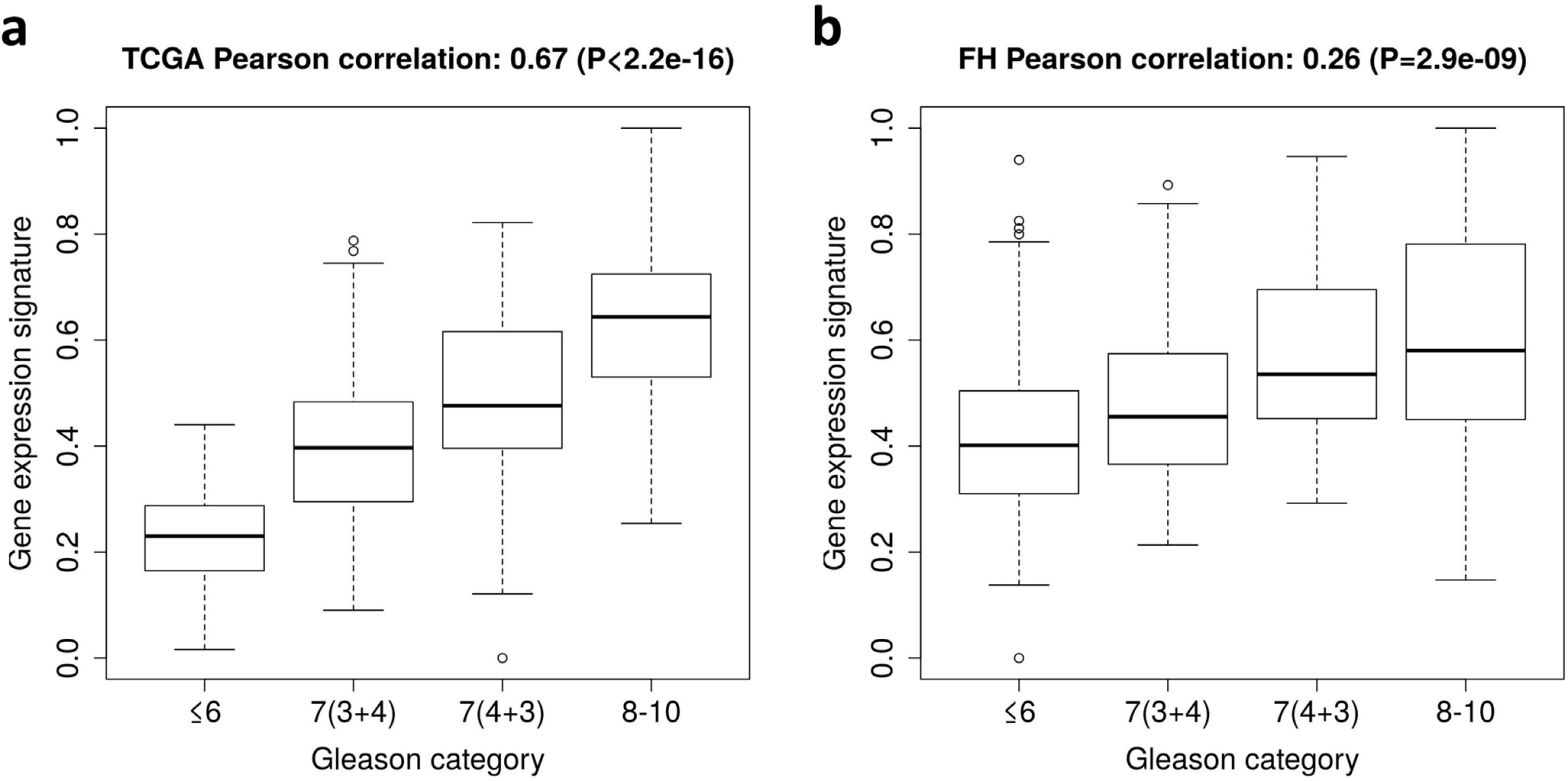
Gene expression signature of Gleason score in The Cancer Genome Atlas (TCGA) dataset and the Fred Hutchinson (FH) cohort **(a)** Box plots of the gene expression signature for patients in different Gleason score categories in TCGA. **(b)** Box plots of the gene expression signature for patients in different Gleason score categories in the FH cohort.

The gene expression signature incorporated 49 genes (Table 2) including *BUB1*, *CENPE*, *CENPF*, *DLGAP5*, *PRC1*, and *SMC4*, which are cell cycle-related genes. As expected, the gene expression signature was strongly correlated with GS in TCGA (Pearson's product-moment correlation = 0.67, P-value <2.2×10^−16^) (Figure 1a). The AUC of the gene expression signature to predict GS high (8-10) vs. low (≤6) tumors was 0.99 in the TCGA dataset.

**Table 2:** Elastic net coefficients of 49 genes in the gene expression signature based on Gleason score (GS)

Gene	Mean expression inGS ≤6	Mean expression inGS 8-10	Difference in mean expression levels	Elastic net coefficient
*PDGFB*	7.5115	8.1313	0.6198	0.1240
*ASPN*	6.4667	8.1400	1.6734	0.1072
*FOXS1*	3.9945	5.4552	1.4606	0.0954
*SMC4*	8.4537	9.3393	0.8856	0.0918
*FAM72B*	3.8462	5.2243	1.3781	0.0889
*ITGBL1*	5.7694	7.8370	2.0676	0.0819
*LPPR4*	3.6591	4.9286	1.2694	0.0741
*SPAG1*	6.9019	7.7345	0.8326	0.0635
*BUB1*	4.8509	6.6930	1.8422	0.0626
*GOLGA7B*	3.7733	5.0629	1.2896	0.0480
*CENPF*	6.6843	8.3503	1.6659	0.0459
*GDF3*	0.5192	1.1654	0.6462	0.0379
*MAPK8IP2*	7.9486	8.8577	0.9091	0.0362
*ESM1*	3.7090	6.2098	2.5007	0.0353
*PRC1*	7.3757	8.4347	1.0590	0.0323
*MYT1*	1.6848	3.6640	1.9792	0.0298
*LRFN2*	1.6411	2.8394	1.1983	0.0286
*SHCBP1*	3.9902	5.5809	1.5907	0.0264
*AHRR*	4.0409	4.8394	0.7985	0.0253
*CBX2*	5.4580	6.9922	1.5342	0.0244
*GMNN*	8.3146	9.1660	0.8513	0.0226
*NUF2*	3.6024	5.3156	1.7133	0.0190
*STC2*	7.1160	8.0827	0.9667	0.0170
*RAI14*	9.0341	9.7371	0.7031	0.0156
*FGF14*	3.9127	5.2610	1.3483	0.0085
*ZNF467*	6.8503	7.9348	1.0845	0.0085
*TMEM132E*	2.2598	3.1011	0.8413	0.0077
*FAM72D*	2.7055	4.0902	1.3847	0.0061
*CST2*	3.6763	5.9120	2.2358	0.0055
*KIF14*	3.2232	4.9544	1.7312	0.0024
*APLNR*	5.3665	6.7831	1.4167	0.0008
*DLGAP5*	4.1281	5.9601	1.8320	0.0008
*CENPE*	5.3055	6.6710	1.3655	0.0002
*IGSF1*	5.0350	3.0068	−2.0282	−0.0039
*NAAA*	11.6720	11.0961	−0.5760	−0.0101
*ASPA*	3.9591	2.4901	−1.4690	−0.0120
*SLC22A1*	2.4821	1.5967	−0.8854	−0.0122
*TAOK3*	11.4033	10.7582	−0.6451	−0.0126
*C2orf88*	6.4390	4.9111	−1.5279	−0.0157
*NCAPD3*	13.3956	11.7854	−1.6103	−0.0173
*GLB1L3*	9.4140	6.5323	−2.8817	−0.0193
*PAGE4*	7.7971	5.2953	−2.5017	−0.0281
*ANO7*	12.2009	10.0601	−2.1409	−0.0312
*EDN3*	5.2927	2.9105	−2.3822	−0.0469
*TPT1*	16.1576	15.6104	−0.5472	−0.0530
*ADPGK*	11.1068	10.5502	−0.5566	−0.0693
*PACSIN3*	10.6533	10.1026	−0.5507	−0.0909
*GLB1L2*	11.9912	10.9522	−1.0390	−0.1905
*PLOD1*	11.6808	11.3165	−0.3644	−0.2418

### Recurrence and metastatic-lethal progression in the FH cohort

The gene expression signature was then calculated in the FH cohort (N=408). The signature was significantly associated with GS (Pearson's product-moment correlation = 0.26, P-value <2.9×10^−9^) (Figure 1b). The AUC of the gene expression signature to predict GS high (8-10) vs. low (≤6) was 0.76.

**Figure 2:**
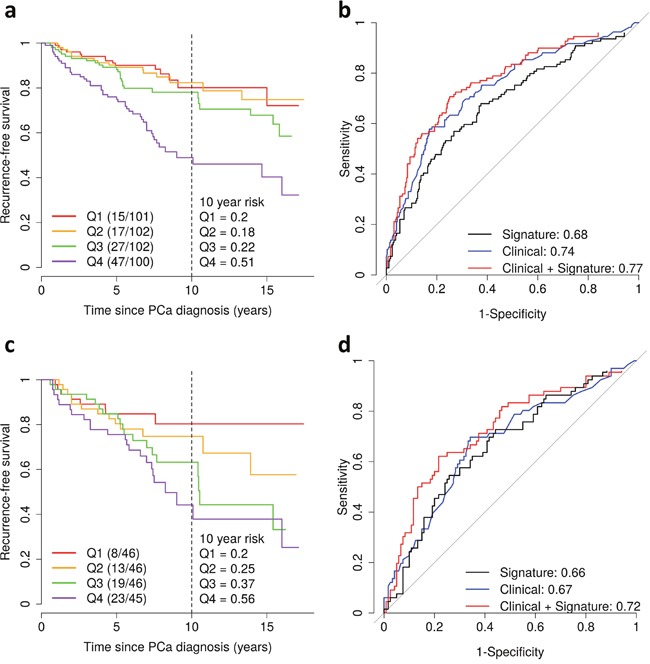
Gene expression signature of Gleason score and prostate cancer recurrence in the Fred Hutchinson cohort **(a)** Kaplan-Meier curves of recurrence-free survival by quartiles (Q1-4) of the gene expression signature. The vertical dashed line shows the recurrence-free survival rate at 10 years after diagnosis. Shown in parentheses are the number of events/number of patients. **(b)** Area under the curve for prediction of recurrence for the gene expression signature alone (black) and for clinicopathological factors (age at diagnosis, Gleason score and pathological stage) with (red) and without (blue) the gene expression signature. **(c-d)** Same analyses as in Figure 2a and b for patients with Gleason score 7 tumors.

We next evaluated risk of recurrence based on the level of differentially expressed transcripts measured by the gene signature. A 25% increase in the gene expression signature was associated with a hazard ratio (HR) for recurrence of 1.65 (95% CI: 1.36, 1.98; P = 2.4×10^− 7^), which remained significant after adjusting for age at diagnosis and pathological stage (HR = 1.51; 95% CI: 1.24, 1.82; P = 2.7×10^−5^) (Table 3; Figure 2). When the analysis was restricted to patients with GS 7 tumors, a 25% increase in the gene expression signature was associated with a HR of 1.44 (95% CI: 1.14, 1.82; P = 0.002), which also remained significant after adjusting for age at diagnosis and pathological stage (HR = 1.38; 95% CI: 1.09, 1.76; P = 0.008).

**Table 3:** Hazard ratios and 95% confidence intervals for prostate cancer recurrence per 25% increase in the gene expression signature based on Gleason score

Patients	Model	Recurrence		
		HR	95% CI	P-value
All	Signature alone ^a^	1.65	1.36, 1.98	2.4×10^−7^
	Multivariate ^b^	1.51	1.24, 1.82	2.7×10^−5^
GS 7	Signature alone ^a^	1.44	1.14, 1.82	0.002
	Multivariate ^b^	1.38	1.09, 1.76	0.008

HR: hazard ratio, CI: confidence interval, GS: Gleason score

^a^ Signature (per 25% increase)

^b^ Adjusted for age at diagnosis and pathological stage

**Figure 3:**
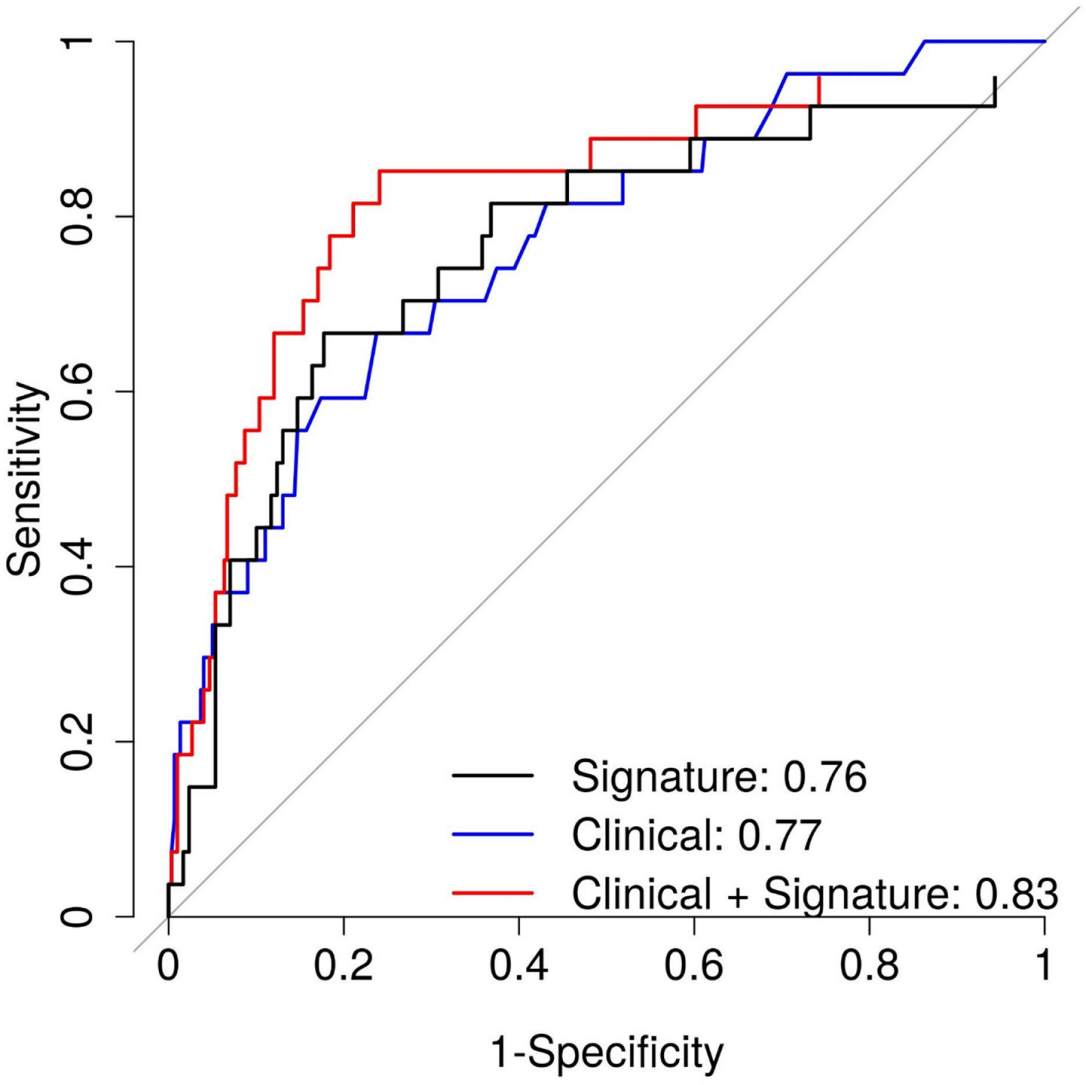
Area under the curve for prediction of metastatic-lethal prostate cancer for the gene expression signature alone (black) and for clinicopathological factors (age at diagnosis, Gleason score and pathological stage) with (red) and without (blue) the gene expression signature

Finally, we estimated the AUC to assess the predictive ability of the gene expression signature. The signature alone had an AUC of 0.68 (Figure 2b) and 0.76 (Figure 3) for predicting recurrence and ML progression, respectively. Adding the signature to a logistic regression model that included clinicopathological factors (i.e., age at diagnosis, pathological stage and GS) significantly improved the goodness of fit of the model for recurrence (likelihood ratio test (LRT) P = 0.0003) and ML progression (P = 0.0004). The AUC increased by 3% and 6% for recurrence (Figure 2b) and ML progression (Figure 3), respectively. In patients with GS 7 tumors, the signature alone had an AUC of 0.66 for recurrence (Figure 2d). When the signature was added to a model with age at diagnosis, pathological stage and GS (3+4 vs. 4+3), the AUC increased by 5% for recurrence and the goodness of fit of the model significantly improved (P = 0.01).

### Gene set enrichment analysis

A GSEA analysis was performed to investigate whether the signature correlates with biological pathways known to be involved in prostate biology and tumor progression. Because the gene signature is based on differentially expressed transcripts in high vs. low GS tumors, higher levels of the signature are expected to reflect biological pathways active in high-grade tumors. We first investigated correlations between the gene expression signature and transcriptome-wide mRNA expression levels. After applying multiple testing correction, gene expression levels of 2,492 genes were significantly correlated with the signature (Bonferroni corrected P-value <0.01). Of the 2,492 genes, 1,372 had transcript levels that were positively correlated with the gene expression signature while 1,120 had transcript levels that were negatively correlated with the signature. GSEA for the 2,492 correlated genes identified gene sets with a FDR q-value <0.05 (Table 4). Higher levels of the signature were associated with increased expression of genes in cell cycle-related pathways including G2M checkpoint, and genes encoding cell-cycle targets of E2F transcription factors, and decreased expression of genes in several pathways including androgen and estrogen response, oxidative phosphorylation, and apoptosis. Several previous studies found evidence that these pathways are important in prostate tumor progression, and the results therefore suggest that our signature captures these relevant biological differences.

**Table 4:** Gene set enrichment analysis results of significant gene sets (FDR Q<0.05) in the gene expression signature

Gene set name	Description	NES
HALLMARK_G2M_CHECKPOINT	Genes involved in the G2/M checkpoint, as in progression through the cell division cycle	2.39
HALLMARK_EPITHELIAL_MESENCHYMAL_TRANSITION	Genes defining epithelial-mesenchymal transition, as in wound healing, fibrosis, and metastasis	2.34
HALLMARK_E2F_TARGETS	Genes encoding cell cycle-related targets of E2F transcription factors	2.05
HALLMARK_ANDROGEN_RESPONSE	Genes defining response to androgens	−3.62
HALLMARK_ESTROGEN_RESPONSE_EARLY	Genes defining early response to estrogen	−2.72
HALLMARK_OXIDATIVE_PHOSPHORYLATION	Genes encoding proteins involved in oxidative phosphorylation	−2.66
HALLMARK_ESTROGEN_RESPONSE_LATE	Genes defining late response to estrogen	−2.56
HALLMARK_PEROXISOME	Genes encoding components of peroxisome	−2.43
HALLMARK_FATTY_ACID_METABOLISM	Genes encoding proteins involved in metabolism of fatty acids	−2.36
HALLMARK_MTORC1_SIGNALING	Genes up-regulated through activation of mTORC1 complex	−2.13
HALLMARK_MYOGENESIS	Genes involved in development of skeletal muscle (myogenesis)	−2.07
HALLMARK_XENOBIOTIC_METABOLISM	Genes encoding proteins involved in processing of drugs and other xenobiotics	−1.84
HALLMARK_ADIPOGENESIS	Genes up-regulated during adipocyte differentiation (adipogenesis)	−1.74
HALLMARK_APOPTOSIS	Genes mediating programmed cell death (apoptosis) by activation of caspases.	−1.73

FDR: false discovery rate, NES: normalized enrichment score

## DISCUSSION

In the present study, we developed a mRNA expression signature of GS using TCGA data, which predicted risk of recurrence and ML progression when tested in an independent patient cohort under long-term follow-up. This transcript signature may improve disease prognostication after radical prostatectomy.

Several previous studies have constructed prognostic gene expression signatures based on cell cycle proliferation genes [3], prostate tumorigenesis related genes [4], and PCa outcomes [5–7]. The cell cycle progression score [3], Prolaris®, was demonstrated to have predictive value for outcomes in RP specimens [14] and needle biopsy [15, 16]. It improved prognosis for biochemical recurrence [3], metastatic progression [17], and PCa death [15, 16]. The gene expression signature based on prostate tumorigenesis related genes [4], Oncotype DX®, improved the prediction of aggressive disease [18] and recurrence [19]. The gene expression signature Decipher™ [5] based on PCa metastasis was also predictive of biochemical recurrence [20], metastasis [21–23], and mortality [24] in independent validation samples.

In addition, several previous studies have identified gene expression signatures based on GS [8–10, 25]. The previous study by Bibikova et al. [8] utilized 512 prioritized genes that were selected based on biological relevance and publicly reported lists of genes differentially expressed in prostate tumors. They constructed a 16-gene signature using genes that were significantly correlated with GS, however, none of them overlaps with the 49 genes in our signature. This may be due to differences in the input gene sets (prioritized genes vs. transcriptome-wide), methods used to construct the signatures (correlation vs. elastic net), and differences in the size of the training datasets (71 samples vs. 333 TCGA samples). Another study by Penney et al., considered 6,100 genes to develop a 157-gene signature of GS (8-10 vs. ≤6) using the adapted nearest neighbor classification method [9]. Out of the 157 genes, five genes overlap with our signature: asporin (*ASPN*), centromere protein F (*CENPF*), endothelial cell specific molecule 1 (*ESM1*), geminin, DNA replication inhibitor (*GMNN*), and PAGE family member 4 (*PAGE4*). Recently, the same group of investigators utilized transcriptome-wide data to generate an updated expression signature of GS that included transcripts from 30 genes [10]. Of those 30 genes, four overlap with our GS signature: anoctamin 7 (*ANO7*), asporin (*ASPN*), galactosidase beta 1 like 3 (*GLB1L3*), and non-SMC condensin II complex subunit D3 (*NCAPD3*). Interestingly, prior evidence also suggests that three of these genes, *ANO7*, *ASPN*, and *NCAPD3*, play a role in PCa progression [26–28]. The *ANO7* gene encodes a prostate-specific cytoplasmic and polytopic membrane protein [29], and its activity is regulated by androgen signaling. Reduced expression of the gene has been associated with high-grade prostate cancers [26], which is consistent with the gene transcript's negative coefficient in our signature of GS. *ASPN* codes for a cartilage extracellular protein, and others have shown that it is over-expressed in both primary tumors and metastatic PCa [27]; this is consistent with the positive coefficient of this gene in our signature. *NCAPD3* plays critical roles in mitotic chromosome assembly and segregation, and its expression has been associated with a decreased risk of recurrence after radical prostatectomy [28]; this is also consistent with the negative coefficient of the gene in our signature. Thus, our gene signature of GS provides confirmation for four of the 30 genes included in the Penney et al. signature for GS.

We did not prioritize the genes based on PCa-related biological pathways or patient outcomes, however, our signature also includes three genes (*CENPF*, *DLGAP5*, and *PRC1*) represented in the cell cycle progression score [3], (Prolaris®), one gene (*ANO7*) in the metastasis-based Decipher™ panel [5], two genes (*ANO7* and *ESM1*) in the PCa recurrence-based signature [6], and two genes (*CENPE* and *CST2*) in the signature for ML progression previously developed by our group [7]. Our GS signature provides confirmation for several of the genes involved in PCa related pathways and some previously highlighted in relation to adverse patient outcomes. In particular, evidence from multiple studies now corroborates the differential expression of *ANO7, ASPN, CENPE*, and *CENPF* in more aggressive PCa.

In addition to alterations in gene expression, other types of molecular biomarkers in prostate tumor tissue may reveal further perturbations in genomic pathways that mediate PCa progression. Thus, the ability to predict aggressive tumor behavior in PCa patients diagnosed with clinically localized disease may be improved by combining different types of genomic signatures. In a previous study from our group, Geybels et al. developed a DNA methylation signature by contrasting high vs. low GS tumors in TCGA and confirmed its value for predicting PCa recurrence in our FH cohort [30]. Of note, our gene expression signature of GS is highly correlated with that DNA methylation signature of GS (Pearson correlation = 0.57). However, in spite of the high correlation, only the *ANO7* gene was highlighted in both signatures.

One limitation of our study is that the gene expression data of the training set (TCGA) and the testing set (FH) were generated using different platforms. RNA-seq data were available in TCGA and array-based data in the FH cohort. In general, RNA-seq read densities are known to be highly correlated with gene expression intensities from microarray measurements [31], and therefore, we used the averaged values without making any transformation. Out of the 49 genes in our signature, 17 (35%) genes had more than one transcript measured in the FH tumor samples. Therefore, we might have missed or oversimplified the differences in gene expression levels from alternative splicing. Another limitation of our study is the small number of cases with metastatic progression or who died of PCa. Since PCa that is clinically localized at diagnosis often takes decades to relapse as metastatic disease or cause disease-specific mortality, a long follow-up period is required to ascertain these outcome events. With a maximum follow-up of 17 years for recurrence (mean of 8 years) in our study, we observed 109 recurrence events including 27 ML events. Therefore, further evaluation of our signature in other PCa cohorts with larger numbers of ML events is warranted.

Gleason score is one of the best predictors of PCa prognosis, however, its utility for assessing prognosis at the time of diagnosis is limited by inter-pathologists variability in grading tumor tissue [32], and the substantial heterogeneity of outcomes in patients with intermediate GS 7 tumors [13]. To better predict subsequent outcomes in PCa patients diagnosed with clinically localized tumors, we developed a gene expression signature based on contrasting high (8-10) vs. low (≤6) GS tumors in TCGA. By focusing on patients in more extreme GS groups, the signature may be less likely to be influenced by inter-pathologists variability in reading GS and may be more predictive of adverse outcomes in patients with intermediate grade tumors. The signature was then tested in a prospectively followed FH-based patient cohort for its ability to predict long-term PCa outcomes. The GS-based gene expression signature significantly improved the prediction of recurrence and ML progression compared to standard clinicopathological parameters. Therefore, the signature may be a useful tool to help improve the prognostication of PCa patients.

## MATERIALS AND METHODS

### Study population

#### The Fred Hutchinson (FH) Cancer Research Center cohort

The FH cohort is composed of male residents of King County, Washington, who were diagnosed with PCa in 1993-1996 [33] or in 2002-2005 [34], ascertained through the Seattle-Puget Sound Surveillance Epidemiology and End Results (SEER) registry, and participated in population-based studies. For this analysis, only the subset of patients who underwent radical prostatectomy as primary treatment and who had gene expression and outcomes data available are included. The Fred Hutchinson Institutional Review Board approved the studies and all participants signed informed consent statements. Clinicopathological information including age at diagnosis, Gleason score, pathological tumor stage (local: pT2, N0/NX, M0; regional: pT3-T4 and/or N1, M0), and prostate-specific antigen (PSA) level at diagnosis was collected from the SEER cancer registry. The participants were followed for recurrence status using detailed patient questionnaires completed in 2004-2005 and 2010-2011, with review of medical records and physician follow-up for clarification as needed. Patients were considered to have recurrence based on the following conditions: (a) post-surgery PSA value of 0.2 ng/mL or greater; or (b) evidence of metastatic progression on a bone scan, MRI, CT, or biopsy; or (c) confirmed PCa-specific death. Vital status and underlying cause of death were obtained from the SEER registry, and cause of death was verified by review of death certificates. Metastatic-lethal (ML) progression was defined based on conditions (b) and (c).

### RNA extraction and profiling

Formalin-fixed paraffin-embedded (FFPE) PCa tumor tissue blocks were obtained from radical prostatectomy samples and used to make H&E stained slides, which were reviewed by a PCa pathologist to confirm the presence and location of prostate adenocarcinoma. For each patient, two 1-mm tumor tissue cores were taken from areas enriched with ≥75% tumor cells from the dominant lesion. The RNeasy® FFPE Kit (Qiagen Inc., Valencia, CA) was used to isolate RNA from tissue cores. RNA samples were quantified with RiboGreen, aliquoted onto 96-well plates and shipped to Illumina for gene expression profiling. Tumor RNA samples from patients with various outcomes were randomly distributed across the plates and laboratory personnel were blinded to this information.

The WG_DASL® HT Assay (Illumina, Inc., San Diego, CA) was used for gene expression profiling. RNA was converted to cDNA using biotinylated oligo (dT) and random nonamer primers, and immobilized to a streptavidin-coated solid support. Prequalification of cDNA was assessed using quantitative RT-PCR and analysis of the housekeeping gene *RPL13a*. Biotinylated cDNAs were annealed to assay-specific oligonucleotides to create PCR templates that were amplified using labeled and biotinylated universal primers. Labeled PCR products were captured on streptavidin paramagnetic beads, washed, and denatured to yield single-stranded fluorescent molecules which were hybridized to the HumanHT-12 v4 Expression BeadChip. Samples were scanned using a BeadArray® Reader that reads the fluorescence intensities, and intensity data file images were extracted for 29,377 transcripts that map to 20,818 genes. Gene expression data were quantile normalized and log2 transformed using R statistical computing software. Low quality probes were filtered out with IlluminaHumanWGDASLv4.db in Bioconductor, leaving 26,051 transcripts for further analysis. Batch effects were removed using ComBat [35]. FH blind duplicate samples from 11 patients that were randomly distributed across the plates had correlations ranging from 0.98 to 0.99, and replicate samples from two patients that were included on every plate had mean correlations of 0.99.

### The Cancer Genome Atlas

The TCGA dataset included 333 PCa patients [36]. Level three RNA-seq data were downloaded from the Cancer Browser (https://genome-cancer.ucsc.edu/), and expression data for 20,530 genes were available.

### Statistical analysis

In the FH cohort, there were 26,051 gene expression probes in 17,923 genes. In case a gene had more than one probe, the average transcript level for that gene was calculated. There were 16,174 genes for which expression data were available in both the FH cohort and in TCGA. All 16K genes were used as input for feature selection using elastic net. Elastic net logistic regression was used to build a gene expression signature of patients with GS 8-10 vs. GS ≤6 tumors in TCGA. The analysis was done using the *glmnet* R package with the binomial family option and an elastic net mixing parameter α equal to 0.5 (α = 0 for ridge regression; α = 1 for lasso regression). Five-fold cross-validation was used to identify the value for lambda that resulted in the highest cross-validation area under the curve (AUC=0.96). The gene expression signature of patient *j* was calculated as follows: ${\text{Geneexpression signature}}_{j}=\;\sum_{i=1}^{n}\alpha_{i}\times E_{\mathrm{ij}}$, where n is the number of genes used to calculate the gene expression signature, $\alpha_{i}$ is an elastic net coefficient of gene *i* and $E_{\mathrm{ij}}$ is a gene transcript level of gene *i* of patient *j*.

In the FH cohort, the correlation of the signature with GS was calculated. Kaplan-Meier curves and Cox proportional hazard models were used to investigate associations between quartiles of the signature and PCa recurrence. Models were adjusted for age at diagnosis and pathological stage. The signature's AUCs for recurrence and ML progression were estimated using the predicted probabilities from logistic regression (*pROC* in R) adjusting for age at diagnosis, pathological stage, and GS ≤6, 7 (3+4), 7 (4+3), and 8-10 groups. A likelihood ratio test was used to evaluate the ability of the signature to improve the prediction of PCa outcomes compared to a model with the clinicopathological parameters age at diagnosis, GS and pathological stage only (*lmtest* in R).

After that, we identified the genes for which the expression levels correlated with the signature (Bonferroni corrected P-value < 0.01). These genes were ranked based on the correlation coefficient and used as input for Gene Set Enrichment Analysis (GSEA) [37]. Gene sets used in the analysis were from the Molecular Signatures Database (MSigDB) hallmark gene set collection, which have been shown to reduce variation and redundancy thereby providing more refined and concise inputs for GSEA [38]. An enrichment score (ES) for each hallmark gene set was calculated, and these scores were normalized to account for the size of the gene set to yield a normalized enrichment score (NES) [37]. A FDR q-value threshold of 0.05 was used.

## Abbreviations

AUC: area under the curve
GSEA: gene set enrichment analysis
FFPE: formalin-fixed paraffin-embedded
FH: Fred Hutchinson
GS: Gleason score
HR: hazard ratio
ML: metastatic-lethal
MSigDB: Molecular Signatures Database
PCa: prostate cancer
PSA: prostate-specific antigen
SEER: Surveillance, Epidemiology, and End Results
TCGA: The Cancer Genome Atlas

## References

[1] Han M, Partin AW, Zahurak M, Piantadosi S, Epstein JI, Walsh PC (2003). Biochemical (prostate specific antigen) recurrence probability following radical prostatectomy for clinically localized prostate cancer. J Urol.

[2] Catalona WJ, Smith DS (1994). 5-year tumor recurrence rates after anatomical radical retropubic prostatectomy for prostate cancer. J Urol.

[3] Cuzick J, Swanson GP, Fisher G, Brothman AR, Berney DM, Reid JE, Mesher D, Speights VO, Stankiewicz E, Foster CS, Moller H, Scardino P, Warren JD (2011). Prognostic value of an RNA expression signature derived from cell cycle proliferation genes in patients with prostate cancer: a retrospective study. Lancet Oncol.

[4] Knezevic D, Goddard AD, Natraj N, Cherbavaz DB, Clark-Langone KM, Snable J, Watson D, Falzarano SM, Magi-Galluzzi C, Klein EA, Quale C (2013). Analytical validation of the Oncotype DX prostate cancer assay - a clinical RT-PCR assay optimized for prostate needle biopsies. BMC Genomics.

[5] Erho N, Crisan A, Vergara IA, Mitra AP, Ghadessi M, Buerki C, Bergstralh EJ, Kollmeyer T, Fink S, Haddad Z, Zimmermann B, Sierocinski T, Ballman KV (2013). Discovery and validation of a prostate cancer genomic classifier that predicts early metastasis following radical prostatectomy. PLoS One.

[6] Shahabi A, Lewinger JP, Ren J, April C, Sherrod AE, Hacia JG, Daneshmand S, Gill I, Pinski JK, Fan JB, Stern MC (2016). Novel gene expression signature predictive of clinical recurrence after radical prostatectomy in early stage prostate cancer patients. Prostate.

[7] Rubicz R, Zhao S, Wright JL, Coleman I, Grasso C, Geybels MS, Leonardson A, Kolb S, April C, Bibikova M, Troyer D, Lance R, Lin DW (2017). Gene expression panel predicts metastatic-lethal prostate cancer outcomes in men diagnosed with clinically localized prostate cancer. Molecular Oncology.

[8] Bibikova M, Chudin E, Arsanjani A, Zhou L, Garcia EW, Modder J, Kostelec M, Barker D, Downs T, Fan JB, Wang-Rodriguez J (2007). Expression signatures that correlated with Gleason score and relapse in prostate cancer. Genomics.

[9] Penney KL, Sinnott JA, Fall K, Pawitan Y, Hoshida Y, Kraft P, Stark JR, Fiorentino M, Perner S, Finn S, Calza S, Flavin R, Freedman ML (2011). mRNA expression signature of Gleason grade predicts lethal prostate cancer. J Clin Oncol.

[10] Sinnott JA, Peisch S, Tyekucheva S, Gerke TA, Lis RT, Rider JR, Fiorentino M, Stampfer MJ, Mucci LA, Loda M, Penney KL (2016). Prognostic utility of a new mRNA expression signature of Gleason score. Clin Cancer Res.

[11] Humphrey PA (2004). Gleason grading and prognostic factors in carcinoma of the prostate. Mod Pathol.

[12] Epstein JI, Zelefsky MJ, Sjoberg DD, Nelson JB, Egevad L, Magi-Galluzzi C, Vickers AJ, Parwani AV, Reuter VE, Fine SW, Eastham JA, Wiklund P, Han M (2016). A Contemporary Prostate Cancer Grading System: A contemporary prostate cancer grading system: a validated alternative to the Gleason score. Eur Urol.

[13] Sakr WA, Tefilli MV, Grignon DJ, Banerjee M, Dey J, Gheiler EL, Tiguert R, Powell IJ, Wood DP (2000). Gleason score 7 prostate cancer: a heterogeneous entity? Correlation with pathologic parameters and disease-free survival. Urology.

[14] Cooperberg MR, Simko JP, Cowan JE, Reid JE, Djalilvand A, Bhatnagar S, Gutin A, Lanchbury JS, Swanson GP, Stone S, Carroll PR (2013). Validation of a cell-cycle progression gene panel to improve risk stratification in a contemporary prostatectomy cohort. J Clin Oncol.

[15] Cuzick J, Berney DM, Fisher G, Mesher D, Moller H, Reid JE, Perry M, Park J, Younus A, Gutin A, Foster CS, Scardino P, Lanchbury JS (2012). Prognostic value of a cell cycle progression signature for prostate cancer death in a conservatively managed needle biopsy cohort. Br J Cancer.

[16] Cuzick J, Stone S, Fisher G, Yang ZH, North BV, Berney DM, Beltran L, Greenberg D, Moller H, Reid JE, Gutin A, Lanchbury JS, Brawer M (2015). Validation of an RNA cell cycle progression score for predicting death from prostate cancer in a conservatively managed needle biopsy cohort. Br J Cancer.

[17] Bishoff JT, Freedland SJ, Gerber L, Tennstedt P, Reid J, Welbourn W, Graefen M, Sangale Z, Tikishvili E, Park J, Younus A, Gutin A, Lanchbury JS (2014). Prognostic utility of the cell cycle progression score generated from biopsy in men treated with prostatectomy. J Urol.

[18] Klein EA, Cooperberg MR, Magi-Galluzzi C, Simko JP, Falzarano SM, Maddala T, Chan JM, Li J, Cowan JE, Tsiatis AC, Cherbavaz DB, Pelham RJ, Tenggara-Hunter I (2014). A 17-gene assay to predict prostate cancer aggressiveness in the context of Gleason grade heterogeneity, tumor multifocality, and biopsy undersampling. Eur Urol.

[19] Cullen J, Rosner IL, Brand TC, Zhang N, Tsiatis AC, Moncur J, Ali A, Chen Y, Knezevic D, Maddala T, Lawrence HJ, Febbo PG, Srivastava S (2015). A biopsy-based 17-gene genomic prostate score predicts recurrence after radical prostatectomy and adverse surgical pathology in a racially diverse population of men with clinically low- and intermediate-risk prostate cancer. Eur Urol.

[20] Alshalalfa M, Crisan A, Vergara IA, Ghadessi M, Buerki C, Erho N, Yousefi K, Sierocinski T, Haddad Z, Black PC, Karnes RJ, Jenkins RB, Davicioni E (2015). Clinical and genomic analysis of metastatic prostate cancer progression with a background of postoperative biochemical recurrence. BJU Int.

[21] Ross AE, Feng FY, Ghadessi M, Erho N, Crisan A, Buerki C, Sundi D, Mitra AP, Vergara IA, Thompson DJ, Triche TJ, Davicioni E, Bergstralh EJ (2014). A genomic classifier predicting metastatic disease progression in men with biochemical recurrence after prostatectomy. Prostate Cancer Prostatic Dis.

[22] Klein EA, Yousefi K, Haddad Z, Choeurng V, Buerki C, Stephenson AJ, Li J, Kattan MW, Magi-Galluzzi C, Davicioni E (2015). A genomic classifier improves prediction of metastatic disease within 5 years after surgery in node-negative high-risk prostate cancer patients managed by radical prostatectomy without adjuvant therapy. Eur Urol.

[23] Karnes RJ, Bergstralh EJ, Davicioni E, Ghadessi M, Buerki C, Mitra AP, Crisan A, Erho N, Vergara IA, Lam LL, Carlson R, Thompson DJ, Haddad Z (2013). Validation of a genomic classifier that predicts metastasis following radical prostatectomy in an at risk patient population. J Urol.

[24] Cooperberg MR, Davicioni E, Crisan A, Jenkins RB, Ghadessi M, Karnes RJ (2015). Combined value of validated clinical and genomic risk stratification tools for predicting prostate cancer mortality in a high-risk prostatectomy cohort. Eur Urol.

[25] True L, Coleman I, Hawley S, Huang CY, Gifford D, Coleman R, Beer TM, Gelmann E, Datta M, Mostaghel E, Knudsen B, Lange P, Vessella R (2006). A molecular correlate to the Gleason grading system for prostate adenocarcinoma. Proc Natl Acad Sci U S A.

[26] Mohsenzadegan M, Madjd Z, Asgari M, Abolhasani M, Shekarabi M, Taeb J, Shariftabrizi A (2013). Reduced expression of NGEP is associated with high-grade prostate cancers: a tissue microarray analysis. Cancer Immunol Immunother.

[27] Ozdemir BC, Hensel J, Secondini C, Wetterwald A, Schwaninger R, Fleischmann A, Raffelsberger W, Poch O, Delorenzi M, Temanni R, Mills IG, van der Pluijm G, Thalmann GN (2014). The molecular signature of the stroma response in prostate cancer-induced osteoblastic bone metastasis highlights expansion of hematopoietic and prostate epithelial stem cell niches. PLoS One.

[28] Lapointe J, Malhotra S, Higgins JP, Bair E, Thompson M, Salari K, Giacomini CP, Ferrari M, Montgomery K, Tibshirani R, van de Rijn M, Brooks JD, Pollack JR (2008). hCAP-D3 expression marks a prostate cancer subtype with favorable clinical behavior and androgen signaling signature. Am J Surg Pathol.

[29] Bera TK, Das S, Maeda H, Beers R, Wolfgang CD, Kumar V, Hahn Y, Lee B, Pastan I (2004). NGEP, a gene encoding a membrane protein detected only in prostate cancer and normal prostate. Proc Natl Acad Sci U S A.

[30] Geybels MS, Wright JL, Bibikova M, Klotzle B, Fan JB, Zhao S, Feng Z, Ostrander EA, Lin DW, Nelson PS, Stanford JL (2016). Epigenetic signature of Gleason score and prostate cancer recurrence after radical prostatectomy. Clin Epigenetics.

[31] Malone JH, Oliver B (2011). Microarrays, deep sequencing and the true measure of the transcriptome. BMC Biol.

[32] Allsbrook WC, Mangold KA, Johnson MH, Lane RB, Lane CG, Amin MB, Bostwick DG, Humphrey PA, Jones EC, Reuter VE, Sakr W, Sesterhenn IA, Troncoso P (2001). Interobserver reproducibility of Gleason grading of prostatic carcinoma: urologic pathologists. Hum Pathol.

[33] Stanford JL, Wicklund KG, McKnight B, Daling JR, Brawer MK (1999). Vasectomy and risk of prostate cancer. Cancer Epidemiol Biomarkers Prev.

[34] Agalliu I, Salinas CA, Hansten PD, Ostrander EA, Stanford JL (2008). Statin use and risk of prostate cancer: results from a population-based epidemiologic study. Am J Epidemiol.

[35] Johnson WE, Li C, Rabinovic A (2007). Adjusting batch effects in microarray expression data using empirical Bayes methods. Biostatistics.

[36] Cancer Genome Atlas Research Network (2015). The Molecular Taxonomy of Primary Prostate Cancer. Cell.

[37] Subramanian A, Tamayo P, Mootha VK, Mukherjee S, Ebert BL, Gillette MA, Paulovich A, Pomeroy SL, Golub TR, Lander ES, Mesirov JP (2005). Gene set enrichment analysis: a knowledge-based approach for interpreting genome-wide expression profiles. Proc Natl Acad Sci U S A.

[38] Liberzon A, Birger C, Thorvaldsdottir H, Ghandi M, Mesirov JP, Tamayo P (2015). The Molecular Signatures Database (MSigDB) hallmark gene set collection. Cell Syst.

